# Exploring the understanding and experience of women with rheumatic diseases regarding fertility intention- a qualitative content analysis

**DOI:** 10.1186/s12905-024-02969-5

**Published:** 2024-02-16

**Authors:** Elham Manouchehri, Mona Larki, Maryam Sahebari

**Affiliations:** 1 Rheumatic Diseases Research Center, Mashhad University of Medical Sciences, Mashhad, Iran; 2 Department of Midwifery, Faculty of Nursing and Midwifery, Mashhad Medical Sciences, Islamic Azad University, Mashhad, Iran; 3 Nursing and Midwifery Care Research Center, Mashhad University of Medical Sciences, Mashhad, Iran; 4 Department of Midwifery, School of Nursing and Midwifery, Mashhad University of Medical Sciences, Mashhad, Iran

**Keywords:** Rheumatic diseases, Content analysis, Fertility intention, Childbearing desire, Pregnancy, Iranian women

## Abstract

**Background:**

Patients with rheumatic diseases (RDs) have a lower desire to have children, fewer children, and a longer interval between their pregnancies, which can be due to the patient’s personal choice, the physician’s advice, changes in sexual activity, changes in fertility, and pregnancy failure. This study aimed to explore the understanding and experience of women with RDs regarding pregnancy intention.

**Method:**

In Mashhad, Northeast Iran, between December 2022 and March 2023, this qualitative inquiry was carried out. Purposive sampling was used to select thirty women with RDs. Semi-structured interviews were used to collect the data. Graneheim and Lundman’s conventional content analysis method was used to analyze the data. The data organization was done using MAXQDA 12 software. Credibility, dependability, confirmability, and transferability have been considered as elements of trustworthiness.

**Results:**

The participants’ data analysis revealed the following main theme: “duality of desire and fear in childbearing”. Five main categories were identified, including “Individual health concerns following pregnancy”, “motherhood and womanhood perceptions”, “concerns about child harm”, “contradictory beliefs and attitudes of significant family members and clinicians about pregnancy”, and “lack of social support for fertility”.

**Conclusion:**

In order to improve the outcomes of pregnancy for women with RDs, the medical professionals who manage them must actively and frequently inquire about their intentions to childbearing and offer them individualized guidance on how to be in the best possible health at the time of conception. Rheumatologists, gynecologists, and reproductive health specialists can better address the sexual and reproductive health needs of this population by enhancing their collaboration in the care of women with RDs.

**Supplementary Information:**

The online version contains supplementary material available at 10.1186/s12905-024-02969-5.

## Introduction

Autoimmune rheumatic diseases (RDs) are characterized by abnormal immune responses, complement activation, cytokine dysregulation, and inflammation [1]. This group of diseases affects women of childbearing age, and its maximum incidence is in the late teens to the fifth decade of life [2]. In these patients, problems related to the disease itself or its treatments can affect fertility, contraception, pregnancy outcomes, and even breastfeeding [3, 4]. In women with RDs, adverse pregnancy outcomes such as pre-eclampsia, pre-term delivery, intrauterine growth restriction, and fetal death are higher than in the normal population [5–9]. Patients with RDs have a lower desire to have children, fewer children, and a longer interval between their pregnancies, which can be due to the patient’s personal choice, the physician’s advice, changes in sexual activity, changes in fertility, and pregnancy failure [10].

The American College of Rheumatology (ACR) has published guidelines related to reproductive health in patients with RDs. In this guideline, screening for pregnancy intention in women with RDs is recommended [11]. Also, the European League Against Rheumatism (EULAR) has published recommendations on family planning, the use of assisted reproductive technology, pregnancy, and menopause in systemic lupus erythematosus or antiphospholipid syndrome in guidelines using the Delphi method [12]. The decision-making process and desire to have children in humans are complex issues that can be influenced by various social, political, and personal factors [13]. The intention to have children is a mental mechanism that is defined as the desire or intention to have children and includes the beliefs and behaviors of a person in this field [14]. In various chronic diseases and different countries, studies have been conducted with a qualitative approach to investigate the motivation, understanding, feelings, and views of the affected people regarding pregnancy, childbearing, and family planning.

Based on the findings of two qualitative studies with the aim of investigating childbearing intentions in women with HIV on antiretroviral treatment, fertility intention in Iranian women with HIV is complex and is influenced by various factors. These factors included the possibility of fetal death, stigma, violence, past pregnancy experiences, infant infection, the health care system, and the patient’s economic status [15, 16].

According to the findings of Armuand et al. (2018) study to investigate the understanding and experience of women and men with cancer regarding childbearing in Sweden, at the time of diagnosis of cancer and during treatment in young patients, it is necessary to consider childbearing counseling in an individual-based manner. The participants in this study expressed concerns about their health during pregnancy, the health status of the fetus, the increased risk of cancer recurrence following pregnancy, and the motherlessness of their child. Because some of these concerns do not have a scientific basis, it is possible to help patients make appropriate decisions about childbearing through counseling, education, and awareness [17].

Pre-conception planning is essential for any person suffering from some kind of chronic disease. In women with RDs, if counseling and preconception planning are not performed, adverse maternal and fetal outcomes may increase following the flare of the disease and drug treatments [18, 19].

Studying and investigating the complex decision-making process for childbearing in the socio-cultural context of Iran in patients with RDs and determining the themes that show the priorities and concerns of women with such diseases regarding childbearing will help the correct decision-making process regarding pregnancy with the least complications or adverse consequences [20].

Several cross-sectional studies have been conducted in some countries [21]. However, there is a need for a qualitative study to investigate the understanding and experience of women with RDs regarding the fertility intention. A qualitative approach allows participants themselves to explain how, why, or what they were feeling and experiencing at a certain time or during an event of interest [22].

Understanding the intention of pregnancy in women with RDs is considered an important issue. Because in this way, fertility counseling, family planning, and improving the health of the patient and her sexual partner, as well as the health of the born children, can be improved [16].

The present study aimed to explore the understanding and experience of women with RDs regarding pregnancy intention.

## Method

### Design

Using a conventional content analysis methodology, the current qualitative study explored the conceptions and experiences of women with RDs regarding pregnancy intentions [23].

### Setting

The study was conducted in 2 public government clinics (Ghaem and Imam Reza hospitals) and two private offices to ensure the greatest diversity in Mashhad, the largest referral city in the east of Iran.

### Participants

Purposive sampling was used to recruit participants for the study. Inclusion criteria included women who were diagnosed with one of the RDs according to the rheumatologist’s diagnosis and were registered in the treatment center (hospital or private office), age between 18 and 45, the ability to get pregnant, effective communication abilities, adequate free time, and a desire to participate in the study.

## Data collection

Data collection started after receiving the Mashhad University of Medical Sciences’ code of ethics. Between December 2022 and March 2023, in four months the data were collected. When the patient visited by the rheumatologist, she was directed to the interview room if she was eligible for the study. The first researcher (EM), a reproductive health researcher with prior experience interviewing women with chronic diseases, conducted all interviews. Interviews were conducted in Persian. After explaining the objectives of the study and obtaining oral and written consent from the participant, questions were asked according to the interview guide. The continued open-ended question in the interview was: Could you please share your experiences with this disease? Could you clarify your intentions toward pregnancy? were some of more precise questions that were asked in the interview. During the interview, probing questions like, “Can you give me an example?” or “Did you mean this….?” were also utilized. It is important to observe that the quantity and arrangement of the questions varied across the interviewees. The questions have been organized according to the participants’ personal experiences, abilities, comprehension of the problem, and the progression of the interviews. Additionally, the researcher took into account non-verbal indicators during the interview process, such as body language and facial expressions. All of the interviews were taped, and then the transcription was done. The total duration of each interview ranged from 40 to 60 min; that depends on the participants’ enthusiasm, verbal proficiency, and patience. When the concepts were thought to be infrequently reproduced or conveyed, data collection terminated, theoretically reaching saturation. 30 married women made up the participants. Each participant was subjected to one interview. Table 1 displays the participants’ profile. Sampling was carried out until the data were saturated. When none of the final three participants produced any new ideas, we knew that the data was saturated.

### Data analysis

All interview sessions took place in a private room within the clinic, depending on the participants’ desires. All interviews were audio recorded, transcriptions were made, and data was entered into MAXQD version 12, which was created and offered by VERBI Software in Berlin, Germany. The initial interview was followed immediately by the data analysis. For analyzing the data, we applied Graneheim and Lundman’s approach [23]. The interviews’ text was initially read several times to get a general idea and feel for its content as a whole. The text of each interview was then broken down into understandable word, phrase, sentence, and paragraph units. The meaning units were condensed and labeled with codes. The content codes were then reviewed for differences and similarities, and similar codes were placed in the initial categories before being turned into subcategories. Finally, as data analysis progressed, subcategories evolved into categories and themes. Two authors of this study (ML and MS) have carried out this process.

### Trustworthiness

In the present study, in order to evaluate the accuracy and quality of the findings, Guba and Lincoln’s four criteria of trustworthiness were used, including credibility, dependability, confirmability, and transferability [24]. Diverse techniques, including long-term engagement strategy, audio recording, verbatim implementation, member assessment of codes, peer review, and sampling until data saturation, were utilized as necessary in this study to assure trustworthiness. Research associates, study advisors, supervisors, and a number of additional observers received the five interviews’ extracted codes and reanalyzed them. These methods, applied in order to secure the concepts and themes, as well as the textual and audio data, were possible to ensure the dependability of the study results. To increase transferability, a clear description of the context, participant selection criteria, and process of data collection, analysis, and reporting is provided so that other researchers can determine whether or not the findings are applicable to their setting. Two experts in qualitative research reviewed the codes and categories obtained from the interviews to ensure the data’s confirmability.

### Ethical considerations

All participants in this study consented to participate after being informed about the study’s objectives, benefits, confidentiality, and anonymity of the information they were about to provide. All participants were given an informed consent form in Persian, the participants’ and researchers’ native language, which they read and signed. In addition to verbal consent, the participants provided written consent. It was also underlined to all women that they might withdraw from the study at any time without consequence. Participants were not required to respond if they did not feel comfortable doing so. In order to minimize “fear, unconscious coercion, and secrecy,” it was made clear to those who participated that their decision to opt out of the study would have no effect on the care they received. All participants were given a book containing information about RDs. Also, the doctor’s visit was free for the patients who were interviewed in the office.

The proposal for this research was approved by the Ethics Committee of Mashhad University of Medical Sciences, Iran, with the code IR.MUMS.IRH.REC.1402.041. Written informed consent was obtained from all participants after explaining the objectives of the research by the first author (EM). Procedures for obtaining informed consent were approved by the above-mentioned institutional ethics committees. All methods were performed in accordance with the relevant guidelines and regulations (Declaration of Helsinki).

## Result

### Participant characteristics

The participants were 30 married women. The ages of the participants ranged from 25 to 40 years.

The RDs of the participants in this study were as follows: five rheumatoid arthritis (16.67%), six systemic lupus erythematosus (20%), three antiphospholipid syndrome (10%), three scleroderma (10%), three Sjogren’s disease (10%), two Behcet’s disease (6.67%), two Sarcoidosis (6.67%), two ANCA associated vasculitis (6.67%), two psoriatic arthritis (6.67%), one granulomatous mastitis (3.33%) and, one ankylosing spondylitis (3.33%).

Eleven of the women (36.6%) had one child, while four women (13.3%) had none. Most of the participants had a high school education or lower (60%). The majority of women were household (83.3%). The characteristics of the participants are presented in Table 1.

The results of the analysis included 234 meaning units, 16 subcategories, 5 categories, and one theme. The main overarching theme emerged as “duality of desire and fear in childbearing”. It consisted of five categories: negative perceptions about personal health following pregnancy; positive perceptions of motherhood and womanhood; concerns about child harm; contradictory beliefs and attitudes of significant others and clinicians about pregnancy; and lack of social support for fertility (Table 2).

### Main theme

The stories of women expressed that they have a perceived need and a positive attitude towards having children, but due to the nature and negative effects that this disease has on pregnancy and vice versa, understanding the consequences of pregnancy, contradictory views from the social network, and the insufficient infrastructure of the health care system make them afraid about getting pregnant. Therefore, women’s experiences are the main actors in the theme of “duality of desire and fear in childbearing”.

1. **Individual health concerns following pregnancy**: The vast majority of the participants indicated their apprehension about becoming pregnant due to the potential adverse implications that pregnancy might have on their own personal health.
  1.1. **Suffering from an ambiguous disease**: All women discussed the difficult aspects of having children. According to them, the condition of variable and unstable health and the signs of progressing disease force them to make an intelligent and considered decision regarding whether or not to have children or increase their current family size.

I became extremely tired and had problems walking, but that wasn’t all: I also experienced painful joints, a fever, a rash that looked like a butterfly, hypersensitivity to the sun, and loss of hair. When it was cold, my fingertips would become white, and I’d get sores in my mouth and nose. If I get pregnant or give birth, it is not clear whether these symptoms will get worse or not. This uncertainty about the condition of the disease bothers me. (P16, systemic lupus erythematosus, 35 years old).

1.2. **Limiting self-care in daily life**: Some women have indicated that they should have a healthy lifestyle; nevertheless, having children creates a barrier to self-care and following through with their treatment procedure. One of the women explained that:

I recognize the importance of leading a healthy lifestyle, which requires adequate sleep and stress reduction. Well, I tell myself, I might not be able to properly care for myself if I become pregnant. I also don’t have enough time to go to the doctor, get tests done, or take my medications as scheduled if I have another baby. That’s why I believe one child is enough. (P20, granulomatous mastitis, 31 years old).

1.3. **Fear of postpartum disease recurrence**: According to the experiences of women who have rheumatoid arthritis diseases, there are periods in which the symptoms become more severe, which are referred to as flare-up periods, and there are periods in which the symptoms improve, which are referred to as regression periods. In other words, they strongly held the belief that the symptoms of the disease changed throughout the course of time. The majority of the individuals pointed out their fear of experiencing another flare-up of their condition after giving birth as their primary motivation for postponing pregnancy. One of the women said:

My medical condition alternates between getting better and getting worse. The physician says that if you become pregnant, the disease will subside, but after childbirth, the condition will be very difficult, so I am doubtful. I am particularly concerned that the rheumatoid arthritis will flare up again after my pregnancy. (P17, Rheumatoid Arthritis, 41 years old).

2. **Motherhood and womanhood perceptions**: From the perspective of women, being a mother was one of the key elements that contributed to the development of a sense of social identity, especially in traditional societies. They held the opinion that becoming a mother was a requirement for women to receive recognition from society because it is a common cultural norm.
  2.1. **Motherhood as an individual desire**: The interviews revealed that having children is one of the interests of women and a worthwhile effort. One participant talked about her experience:

I have two children; my boys are very smart. Despite the fact that I am always around them, I still feel as though I need more kids. (P9, scleroderma, 38 years old).

2.2. **Motherhood as an asset and opportunity to affirm womanhood**: The majority of women placed a high value on motherhood, which was entrenched in cultural and attitudinal factors as well as the restructuring of cultural policy-making bodies to increase the population. This emerged clearly from the words of one of the women, which are as follows:

I typically feel bad about not having been able to have children and not being able to get pregnant. Our country encourages people to have children. I even thought about surrogacy or adoption because of this. (P25, Behcet’s disease, 41 years old).

The narratives of women participating in the research expressed the concept of identity completion after becoming mothers. They believed that the sense of completeness has a close connection with the category of motherhood, and because they consider motherhood to be effective in establishing their identity, they feel pleasure and fulfillment from motherhood. One woman shared her experiences as follows:

Now when I want to wash the baby, it’s difficult for me, but I can cope with it because a mother’s sweetness is excellent and delightful. It somehow makes femininity complete because I am proud to be a mom (P22, Rheumatoid arthritis, 32 years old).

2.3. **Regret for missing the opportunity of motherhood**: Women stated that their age had increased and that the chance of becoming pregnant had decreased because of their medical conditions. Some of them felt bad about this process. One of the women spoke up:

Now my age is increasing and my pregnancy is getting late. I sometimes think to myself that the disease continues to prevent me from having children, and regret remains for me. (P2, Rheumatoid arthritis, 32 years old).

3. **Concerns about child harm**: The women believed that rheumatoid arthritis has an impact on a variety of aspects of life, including one’s occupation, leisure activities, and social interactions. They believed that they were faced with not being able to meet a newborn’s numerous demands, such as preparing food, nursing the infant, cleansing the infant, and creating a hygienic environment. Women thought that they were not capable of becoming adequate mothers since they were unable to complete a significant number of the tasks. This concern was so great that some of the participants decided against having children.
  3.1. **Fear of congenital abnormality**: Concerns about potential harm to the fetus were one major driving force behind avoiding pregnancy. One of the participants said:

I got pregnant once. My baby was born at seven months due to pregnancy poisoning and died 48 h later. Well, I am afraid that the experience will repeat itself and the child will be injured. For example, I read in the articles that the child may have a cleft lip or six fingers. (P16, scleroderma, 42 years old).

3.2. **Failure to meet the infant’s requirements**: One of the concerns that the women had was that the disabilities brought on by their condition would prevent them from being able to respond to and meet the requirements of their child in a way that was suitable.

I worry that I won’t be able to take care of the baby. Or that I’m not sufficiently competent to take care of my child. Because this illness caused me a lot of trouble, including severe joint pain. (P11, sarcoidosis, 27 years old).

According to the experiences of women, rheumatoid arthritis has a variety of negative impacts on one’s physical health and can lower the standard of care provided to children because this disease results in pain, low energy levels, and fatigue. One of the women described her experience like this:

I am a mother. I cannot play with my child. And because I’m so exhausted, I feel like he’s kind of losing out on that interaction. I believe that if I become pregnant, this problem will worsen, and my unborn child will suffer from this issue. (P22, Rheumatoid arthritis, 32 years old).

4. **Contradictory beliefs and attitudes of significant family members and clinicians about pregnancy**: According to the statements made by women, having children while suffering from rheumatoid arthritis is not a simple task and is fraught with a number of potential difficulties. On the other hand, some significant people in the lives of women and health care providers were in favor of having children, while others were opposed to the idea.
  4.1. **Husband’s reluctance to have offspring**: The majority of women interviewed stated that their husbands consider the advantages and disadvantages of having children before deciding that they should not have any more children because the mother’s physical health is at risk. The quote of one of the women was that:

My husband interacts with me and attempts to reduce my anxiety and stress regarding the pregnancy. He desires for me to become pregnant on the condition that I am not injured. (P29, necrotizing spondylitis, 40 years old).

4.2. **Prohibition of pregnancy by clinicians**: Women have said that the majority of medical professionals have advised them that they are not allowed to become pregnant because of the risks that it creates for them. One of the women raised this point of view:
  Due to vasculitis, my physician prohibited pregnancy in any situation. She told me, “One kid you have is sufficient”. Pregnancy carries a significant danger. (P12, vasculitis, 38 years old).

4.3. **Relatives’ tendency towards creating offspring**: Some women have stated that their families actively encourage them to have children, despite the fact that they have been diagnosed with a disease and their family members are aware of this reality. One of the women whose mother had supported her decision to become pregnant and urged her to do so related her story as follows:

My mom really wants me to have another child. She says that every child keeps a few illnesses from their mother. Honestly, I was also influenced to get pregnant again. (P26, Behcet’s disease, 37 years old).

5. **Lack of social support for fertility**: Social support from the people around them, especially the parents of the couple, other family members, and their network of friends, includes emotional support, confidence in helping to take care of the child, and solutions to their worries about having a child. This was despite the fact that the majority of women did not benefit from social support to take care of their children.
  5.1. **Neglecting the need to fertility by health care providers**: With regard to the experiences of women, the priority of specialists is to control the disease and prevent its progression, and they do not give enough attention to the concerns of having children. One of the participants stated:

My physician seems to be of the opinion that prevention of disease progression is more important than my pregnancy at the moment…( P2, Rheumatoid arthritis, 32 years old).

5.2. **Insufficient support from healthcare providers**: The women stated that the health care system and the health care providers do not have a specialized program for women who are suffering from diseases during pregnancy and after childbirth to make this period easier for them. One woman explained:

I was pregnant with twins. The physical challenges of giving birth, plus the mental and emotional demands of caring for a newborn, are bound to leave me feeling exhausted. However, the healthcare system does not offer women in the same situation as me any assistance. For instance, if I am sick, the health care provider comes to visit the kid at my house. (P5, Sjogren’s disease, 38 years old).

Women who took part in this research said that their physicians did not inquire about their intentions to have children or increase them. As a direct consequence of this, they came to the conclusion that they needed to repress this desire because they should not necessarily have the desired number of children.

I’m not asked about pregnancy by my doctor, but I used to take too many drugs. Therefore, I read that these pills and ampoules are toxic during pregnancy. I have done a lot of research on the Internet on this pill’s impact on the fetus while pregnant. One individual commented, for instance, “There is no problem,” and another replied by saying “There is a problem, which is why I am very stressed out.” These inquiries cannot be made since the healthcare professional is pressed for time. (P11, sarcoidosis, 27 years old).

5.3. **The requirement for additional support from family and husband**: Women who have rheumatoid arthritis struggle with a variety of limitations and disabilities, which may cause them to require additional help from their families and husbands during pregnancy and after childbirth. If they are deprived of it, it will lead to their not intending to get pregnant. One of the women’s experiences provides insight on this topic, and it is as follows:

My mother is ill and unable to assist me, and I am without assistance for taking care of my kid. I ask myself, “How can I care for my child by myself after giving birth?” I cannot rely on anyone for assistance. (P13, rheumatoid arthritis, 35 years old).

5.4. **Misunderstanding of healthcare staff about pregnancy risks and benefits**: The majority of the women noted that healthcare workers did not have accurate information regarding their condition, and the majority of the time, they did not provide clear answers to the questions that the women asked in the health sector. Because of this issue, there is a reasonable possibility that women will ignore their decision to have more children. They stated that physicians’ and caregivers’ lack of awareness is one of the most challenging aspects of living with a chronic illness. One of the women said about her experience: Some workers are ignorant about my disease in the health care system. When I explain that I have a form of rheumatic disease, the medical staff is not usually understanding. The fact that they are not aware of my medical condition is something that I struggle with a lot. (P18, Sjogren’s disease, 33 years old).
5.5. **Poor patient-provider communication during consultation about pregnancy**: Many women felt that professionals did not communicate well with them when discussing their pregnancy decisions. One of them said:

The doctor’s office is so busy that they sometimes lack attention or are bored when I desire to inquire about matters other than my illness. They don’t pay much attention when it comes to pregnancy questions. Therefore, I am embarrassed to ask my questions about pregnancy in this condition. (P13, rheumatoid arthritis, 35 years old).

**Table 1:** Characteristics of the study participants (N = 30)

Variables			Mean ± SD	Frequency	Percent
Age (years)	Participants		34.8 ± 5.12		
	Spouses		40.04 ± 6.03		
Education	Participant	Diploma and below		20	66.67
		Bachelor of Science		8	26.67
		Master of Science and higher		2	6.67
	Husband	Diploma and below		18	60
		Bachelor of Science		6	20
		Master of Science and higher		6	20
Occupation	Participant	Employee		2	6.67
		Self-employment		3	10
		Household		25	83.33
	Husband	Employee		7	23.33
		Self-employee		14	46.67
		Laborer		9	30
No. of Children	0			4	13.33
	1			11	36.67
	2			9	30
	3			6	20
No. of Abortion	0			18	60
	1			9	30
	2			3	10
Place of Interview	Public clinic			16	53.33
	Private office			14	46.67

**Table 2:** Theme, categories, and subcategories emerged from the interviews

Sub categories	Categories	Theme
Suffering from an ambiguous disease	Individual health concerns following pregnancy	Duality of desire and fear in childbearing
Limiting self-care in daily life		
Fear of postpartum disease recurrence		
Motherhood as an individual desire	Motherhood and womanhood perceptions	
Motherhood as an asset and opportunity to affirm womanhood		
Regret for missing the opportunity of motherhood		
Fear of congenital abnormality	Concerns about child harm	
Failure to meet the infant’s requirements		
Husband’s reluctance to have offspring	Contradictory beliefs and attitudes of significant family members and clinicians about pregnancy	
Prohibition of pregnancy by clinicians		
Relatives’ tendency towards creating offspring		
Neglecting the need to fertility by health care providers	Lack of social support for fertility	
Insufficient support of healthcare providers		
The requirement for additional support from family and husband		
Misunderstanding of healthcare staff about pregnancy risks and benefits		
Poor patient-provider communication during consultation about pregnancy		

## Discussion

In the present study, using a qualitative method, fertility intention was investigated in women with RDs, who has a good condition for pregnancy. The main theme that emerged from the analysis of the participants’ interviews was “duality of desire and fear in fertility”.

In several studies conducted in different countries, women who have chronic diseases share comparable worries about sexual and reproductive health issues that are specific to their medical conditions, among which RDs, cystic fibrosis, type 1 diabetes, epilepsy, and MS can be mentioned [25–29].

According to the results of a study using the nominal group technique to determine research priorities for supporting women with RDs, one of the most important topics was pre-conception, pregnancy, and post-natal counseling and care. The lay and professional stakeholder groups came to a consensus that research with a high priority was needed to enhance shared decision-making in healthcare, high-quality conversations during the pre-conception stage, evidence-based information on medication use during pregnancy and breastfeeding, and more individualized approaches to care [30]. In other words, it is possible to make appropriate decisions about fertility and childbearing by providing accurate, evidence-based, and high-quality information [30].

One of the categories obtained in this study was “individual health concerns following pregnancy”. According to participants, focusing on pregnancy may limit their self-care. Also, the more severe recurrence of the disease in the postpartum period and the lack of adequate and appropriate information in this field were among their concerns. In other studies that have been conducted to explore the childbearing perspectives of women with type 1 diabetes and CF, participants believed that being pregnant could affect their self-care routine [25, 26]. Aside from having practical worries about getting pregnant, women also want additional information about how pregnancy can alter their chronic disease and the symptoms related to it [27]. Women’s information demands seem to have two directions: they want to know how their chronic disease will affect their preconception well-being, pregnancy, and parenthood, as well as how pregnancy will affect their chronic condition [31].

In the “motherhood and womanhood perceptions” category, the participants of the present study mentioned their concerns about motherhood itself or motherhood as a manifestation of being a woman. In other words, they considered their status as women largely dependent on having children and being a mother [32]. Some religions, like Islam, advocate procreation and have established accepted rules about it [33]. In addition to enhancing women’s perception of identity, motherhood also fulfills marital needs like carrying on the family line from husbands and in-laws [25, 34].

One of the important categories obtained in the present study was “concerns about child harm”. Almost all the participants expressed concern about the effect of the disease or drugs on the fetus and the possibility of a similar disease in their fetus. They also mentioned the lack of facilities for taking care of newborns as one of the reasons for their hesitancy about childbearing.

Numerous participants with chronic diseases in qualitative studies emphasized the significance of evaluating their health before and after planning to get pregnant, as well as the effects of their health on future offspring. They were concerned that issues like prenatal abnormalities, preterm birth, or stillbirth would affect their fetuses [25, 26]. A mixed methods study by Ackerman et al., revealed that the most important information that women with RDs searched for was the effect of the disease and the toxicity of their medication during pregnancy and breastfeeding [35].

The category “contradictory beliefs and attitudes of significant family members about pregnancy” shows the significant impact of the opinions of spouses and other relatives on the participants’ fertility desires. In most cases, husbands of affected women were willing to have a new child if they were relatively sure that there would be no adverse consequences for the mother and the fetus. But the reactions of other relatives were different, from recommending not to have children to emphasizing having children despite the possibility of adverse outcomes the mother and the fetus.

Other studies have also shown that male partners and families have an impact on a woman’s decision to become pregnant. A loss of social position, rejection, and stigma may follow childlessness [25, 36]. Studies on the desire for childbearing in HIV-positive women in Mozambique and Iran demonstrate how the social and cultural context has a significant impact on childbearing customs. Some participants claimed that they felt pressured by family members, such as parents. For some of the participants, their spouse made the choice, disregarding the wife’s opinions [16, 34].

The category “lack of social support for fertility” demonstrates how little attention healthcare professionals have paid to the needs of fertility in women with RDs. Even the spouses and families of the participants do not provide enough support in this regard.

In some qualitative studies on patients with chronic diseases, this lack of support was described as violence in the hospital, lack of support from spouse and family, stigmatization, and a lack of support services and insurance [16, 34]. In the study of Chew et al., women with rheumatoid arthritis received a sense of support from membership in online peer groups [27].

According to the findings of the present study, the participants stated that there is no suitable reference for obtaining information about the outcomes and risks of pregnancy. It seemed that the medical staff and healthcare providers also did not have enough information. On the other hand, crowding and insufficient time for visiting patients often cause counseling about fertility and childbearing to be neglected. Of course, sometimes the difficulty in communicating between the patient and the specialist or healthcare provider makes them refrain from entering into these issues.

The interaction between women and their healthcare professionals, which is essential to achieving successful care, affects engagement with pregnancy counseling. Participants discussed unpleasant experiences they had with their doctors, which affected the degree to which they disclosed their plan to become pregnant. Women’s anxiety and shame were made worse by service providers with dominant attitudes who did not help them meet their expectations [25, 26, 31, 35–37].

In line with our findings, patients with other chronic illnesses believed that healthcare providers lacked sufficient time to address such a sensitive subject, and the time constraints made it impossible for many participants to discuss sexual matters [38, 39]. Healthcare providers must effectively interact with women in order to create treatment plans that are individualized or condition-specific to their requirements throughout pregnancy. Inadequate staff training and inadequate resources may make the healthcare system’s lack of a holistic strategy more difficult to address [25, 26, 35, 40]. Providing reproductive and sexual health care and counseling for these people is an important part of comprehensive health care for these women [39].

This study has several limitations. The qualitative nature of this study means that it is not generalizable. Also, we explored pregnancy intention in women with RDs therefore, the transferability of these results to communicable diseases is not acceptable.

Despite these limitations, qualitative methods allowed for a more in-depth exploration of patient experiences and preferences than quantitative methods. Participants in this study represent regional diversity as a result of the fact that we selected them from both private offices and public centers. However, we considered the maximum variation in sampling. This is the first qualitative study to explore the particular preferences of Iranian women with a variety of RDs about their fertility intentions.

## Conclusion

In order to improve the outcomes of pregnancy for women with RDs, the medical professionals who manage them must actively and frequently inquire about their intentions to childbearing and offer them individualized guidance on how to be in the best possible health at the time of conception. Rheumatologists, gynecologists and reproductive health specialists can better address the sexual and reproductive health needs of this population by enhancing their collaboration in the care of women with RDs.

### Electronic supplementary material

Below is the link to the electronic supplementary material.

**Supplementary Material 1:** Fertility Intention in Women with RDs

## Data Availability

The datasets used and/or analyzed during the current study are available from the corresponding author on reasonable request.

## Abbreviations

RDs: Rheumatic Diseases
ACR: American College of Rheumatology
EULAR: European League Against Rheumatism
HIV: Human Immunodeficiency Virus
ANCA: Antineutrophilic cytoplasmic antibody
CF: Cystic Fibrosis
