# Supplementary material for: Exploring the understanding and experience of women with rheumatic diseases regarding fertility intention- a qualitative content analysis
Source: BMC Womens Health. 2024 Feb 16;24:124. doi: 10.1186/s12905-024-02969-5 (PMC10874060; doi:10.1186/s12905-024-02969-5)
Supplement: Supplementary file 1 — Supplementary Material 1: Fertility Intention in Women with RDs [file 12905_2024_2969_MOESM1_ESM.docx]

**Exploring the Understanding and Experience of Women with Rheumatic Diseases Regarding Fertility Intention- A qualitative content analysis**

**Interview Guide:**

- What do you know about your rheumatic disease?
- What do you know about the effect of your disease on fertility?
- Please share your experiences, thoughts and feelings about pregnancy.
- Has your disease affected your intention to get pregnant? How?
- Do you have any concerns about this?
- What do you think about pregnancy and motherhood despite this disease?
- What other issues have affected your desire to get pregnant?
- Has your family planning for having children been affected by this diagnosis? Why?
- What information do you need about your reproductive health?
- How do you search for reproductive health information?
- From which source do you prefer to get the information you need about your reproductive health?
- Do you have experience with a doctor or health care provider talking to you about your pregnancy and your intentions?
- Please describe this experience.
- What does your family think about preparing for pregnancy?
- Is there anything else you would like to say about your childbearing and reproductive health?

**Probing questions:**

- Please explain more.
- You mean that....?
- Can you explain what you mean by this sentence more precisely?
